# Targeting Endoplasmic Reticulum Stress with Tauroursodeoxycholic Acid: A Potential Therapeutic Strategy for Allergic Airway Diseases

**DOI:** 10.1159/bmh/aboag006

**Published:** 2026-10-10

**Authors:** Yining Han

**Affiliations:** School of Medicine, The Chinese University of Hong Kong, Shenzhen, China

**Keywords:** Tauroursodeoxycholic acid, Allergic airway disease, Endoplasmic reticulum stress, Unfolded protein response, Therapeutic strategy

## Abstract

**Background:**

Allergic airway diseases (AADs) such as asthma and allergic rhinitis impose a heavy global health burden, characterized by chronic airway inflammation, airway hyperresponsiveness, and structural remodeling. Endoplasmic reticulum (ER) stress has been increasingly identified as a key driver of AAD pathogenesis. As a well-characterized chemical chaperone, tauroursodeoxycholic acid (TUDCA) exhibits promising therapeutic potential for AADs by regulating immunity, relieving inflammation, and reversing airway pathological lesions.

**Summary:**

This review summarizes how ER stress drives AAD development and progression of AADs and details the molecular mechanisms underlying the therapeutic effects of TUDCA by targeting ER stress-related unfolded protein response signaling pathways. Additionally, it highlights preclinical evidence supporting the efficacy of TUDCA and discusses the prospects and challenges associated with its clinical translation.

**Key Messages:**

ER stress is a key pathogenic factor in AADs; TUDCA can target ER stress-related signaling pathways to exert therapeutic effects on AADs; preclinical studies confirm the efficacy of TUDCA, and its clinical translation may provide a novel ER stress-targeted strategy to optimize AAD management and improve patient outcomes.

## Introduction

Allergic airway diseases (AADs) are a group of complex immunological disorders, primarily allergic asthma and rhinitis. AAD prevalence has risen sharply worldwide in recent decades; around 350 million individuals live with asthma globally, posing a severe public health challenge [1–4]. AAD is characterized by chronic inflammation predominantly driven by T helper 2 (Th2) immune responses. These conditions are initiated and exacerbated by exposure to environmental allergens, leading to airway hyperresponsiveness (AHR), mucus hypersecretion, and airway remodeling [5–7]. AAD pathophysiology relies on complex crosstalk among multiple cell populations: airway epithelial cells, immune cells (mast cells, eosinophils, T lymphocytes), and structural cells (fibroblasts, airway smooth muscle cells [ASMCs]) [8–11]. Emerging evidence highlights the pivotal role of cellular stress responses, particularly endoplasmic reticulum (ER) stress, in modulating these pathological processes [12–14]. Notably, ER stress engages in crosstalk with oxidative stress and mitochondrial dysfunction to form a vicious cycle that perpetuates inflammation and tissue injury [14, 15].

Given the well-documented contribution of ER stress to key pathogenic processes in AADs, targeting this pathway represents a promising therapeutic avenue. Tauroursodeoxycholic acid (TUDCA), a hydrophilic bile acid derivative, has emerged as an effective chemical chaperone capable of alleviating ER stress by enhancing protein folding and reducing unfolded protein response (UPR) activation. Initially applied to treat hepatobiliary disorders, TUDCA exerts protective effects across multiple ER stress-related disease models, such as neurodegeneration, retinal degeneration, and renal fibrosis [16–18]. Mechanistically, TUDCA modulates key ER stress sensors, such as inositol-requiring enzyme 1α (IRE1α), protein kinase R-like ER kinase (PERK), and activating transcription factor 6α (ATF6α), and downstream apoptotic pathways, thereby restoring cellular homeostasis and attenuating inflammation [16, 17]. Recent preclinical animal studies have begun to elucidate the potential of TUDCA in AADs [19, 20]. To date, no published clinical studies have yet evaluated the effects of TUDCA in patients with AADs. TUDCA, as a potent ER stress modulator and chemical chaperone, holds promise as a novel therapeutic agent capable of targeting these fundamental pathogenic processes. This review aimed to systematically delineate the current understanding of ER stress involvement in AADs and to comprehensively evaluate the emerging evidence supporting TUDCA’s therapeutic potential, thereby providing a theoretical foundation for the development of innovative treatment strategies.

## The Role of ER Stress in the Pathophysiology of AADs

### Molecular Mechanisms of ER Stress

The ER is a pivotal organelle in eukaryotic cells that is responsible for protein synthesis, folding, post-translational modification, and intracellular trafficking. Intrinsic or extrinsic stressors (hypoxia, oxidative stress, viral infection, and allergen exposure) disrupt ER homeostasis, leading to the accumulation of unfolded or misfolded proteins within the ER lumen. This condition, termed ER stress [14, 20–23], activates an intricate signaling network known as the UPR. The UPR is mediated by three primary sensors: IRE1α, PERK, and ATF6α (shown in Fig. 1) [24–28]. Under physiological conditions, all three sensors are maintained in an inactive state through direct association with the chaperone-binding immunoglobulin protein (BiP, also known as glucose-regulated protein 78, GRP78). Upon detection of misfolded proteins, BiP exhibits higher affinity for these aberrant proteins, leading to its dissociation from IRE1α, PERK, and ATF6. This dissociation permits the independent activation of three distinct signaling cascades, which collectively result in the restoration of ER homeostasis by attenuating protein synthesis, enhancing protein degradation, and upregulating chaperone expression to facilitate proper protein folding within the ER lumen [24–28].

**Fig. 1.**
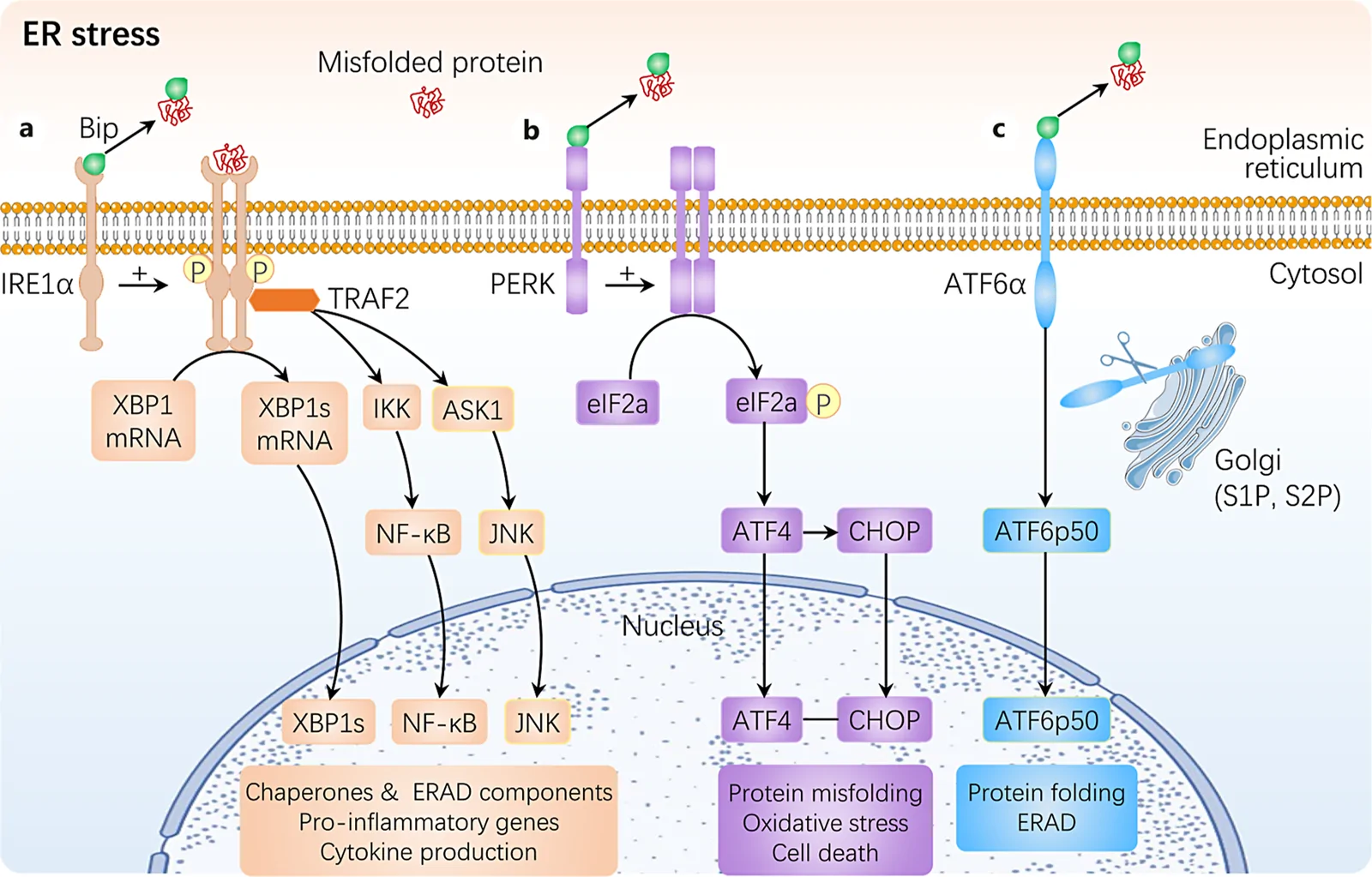
Classic unfolded protein response (UPR) signaling pathways activated by endoplasmic reticulum (ER) stress. **a** Shows activated IRE1α catalyzes XBP1 mRNA splicing and initiates inflammatory signaling via TRAF2. **b** Displays PERK-mediated eIF2α phosphorylation, translational suppression and ATF4/CHOP induction. **c** Depicts trafficking and proteolytic cleavage of ATF6α within the Golgi apparatus to generate active ATF6 p50, which enters the nucleus to upregulate ER homeostatic genes.

#### IRE1α-XBP1 Axis

IRE1α is a type I transmembrane protein that possesses an N-terminal serine/threonine kinase domain and a unique RNase domain in its cytoplasmic region. Following BiP dissociation during ER stress, IRE1α undergoes oligomerization and autophosphorylation, which activates its RNase domain. The activated RNase targets the mRNA encoding X-box binding protein 1 (XBP1), removing a 26-base-pair intron to generate the active transcription factor spliced XBP1 (XBP1s). XBP1s translocates to the nucleus to upregulate genes encoding proteins that increase ER protein-folding capacity and assist in the degradation of misfolded proteins by ER-associated degradation (ERAD) [25, 29]. Notably, IRE1α-mediated XBP1 splicing in response to Toll-like receptor (TLR) ligation has been demonstrated to be crucial for cytokine production [30]. Beyond protein homeostasis regulation, phosphorylated IRE1α also drives pro-inflammatory signaling when ER stress persists. Phosphorylated IRE1α recruits the adapter tumor necrosis factor receptor-associated factor 2 (TRAF2), forming an IRE1α-TRAF2 complex that activates IκB kinase (IKK) and apoptosis signal-regulating kinase 1 (ASK1). Activated IKK degrades the NF-κB inhibitory protein IκB, allowing nuclear factor-κB (NF-κB) to enter the nucleus and initiate transcription of pro-inflammatory cytokines. Meanwhile, active ASK1 phosphorylates and activates c-Jun N-terminal kinase (c-JNK). Together, the IRE1α-TRAF2-NF-κB/JNK axis creates a direct molecular bridge linking sustained ER stress to inflammatory signaling [30–32].

#### PERK-eIF2α-ATF4/CHOP Pathway

PERK is a second ER transmembrane sensor kept silent by BiP binding under basal conditions. Once ER stress separates BiP from PERK, PERK oligomerizes and undergoes autophosphorylation to activate its cytoplasmic kinase domain. Activated PERK phosphorylates the alpha subunit of eukaryotic translation initiation factor 2α (eIF2α), a core protein required for initiating all cytoplasmic mRNA translation [33, 34]. Phosphorylation of eIF2α potently attenuates global mRNA translation, thereby reducing the influx of newly produced polypeptides into the overloaded ER and temporarily easing ER’s folding burden, which constitutes the adaptive protective function of PERK activation in early ER stress.

However, this global translational arrest paradoxically enhances the selective translation of a subset of stress-responsive mRNAs, notably activating transcription factor 4 (ATF4). ATF4 functions as a master regulator of the integrated stress response, inducing genes involved in amino acid metabolism, redox balance, and autophagy. Under chronic ER stress, sustained ATF4 activity drives the transcription of C/EBP-homologous protein (CHOP) [35–37]. ATF4 and CHOP form heterodimers that promote apoptosis by increasing protein synthesis, thereby exacerbating protein misfolding, oxidative stress, and cell death [23]. ATF4-CHOP-driven, autonomous apoptosis likely evolved to eliminate severely stressed, damaged, or infected cells within the organism. Therefore, small-molecule agents that reduce protein synthesis or specifically modulate the PERK-eIF2α-ATF4/CHOP axis hold therapeutic potential for treating protein misfolding disorders [35, 38].

#### ATF6 Proteolytic Activation

ATF6α is a type I transmembrane cAMP response element-binding protein family transcription factor anchored on the ER membrane at rest, with its transcriptionally functional domain retained in the ER lumen and sequestered by BiP. During ER stress, BiP dissociates from ATF6α, enabling ATF6α to translocate to the Golgi apparatus via vesicular transport. Inside the Golgi, sequential cleavage by site-1 protease (S1P) and site-2 protease (S2P) releases the active N-terminal fragment ATF6p50, which enters the nucleus to upregulate genes that expand ER capacity, improve protein folding, and promote ERAD-mediated clearance of misfolded proteins [25, 38, 39].

The UPR can be divided into adaptive and maladaptive phases. Adaptive UPR relieves ER stress via upregulating chaperones, boosting ERAD and restoring calcium balance to restore cellular homeostasis. By contrast, sustained unresolved ER stress switches UPR to a maladaptive state: IRE1α-XBP1 and PERK-eIF2α-ATF4/CHOP signaling trigger pro-inflammatory cytokine secretion, ROS buildup, and proapoptotic gene transcription, eventually leading to irreversible cell damage and death. This adaptive-to-maladaptive transition is central to the pathogenesis of allergic airway diseases. Oxidative stress directly disrupts ER proteostasis via ROS, which induces the accumulation of misfolded proteins and depletes Ca^2+^ stores in the ER, further exacerbating ER stress. As a result, ER stress and oxidative stress form a self-amplifying vicious cycle that continuously aggravates cellular dysfunction and tissue inflammation across multiple chronic inflammatory diseases, including allergic airway diseases [40].

### ER Stress as a Driver of AADs

Compelling evidence implicates ER stress and dysregulated UPR signaling as critical drivers of the pathogenesis of AADs, contributing to epithelial dysfunction, immune dysregulation, and airway remodeling (shown in Fig. 2) [37, 41].

**Fig. 2.**
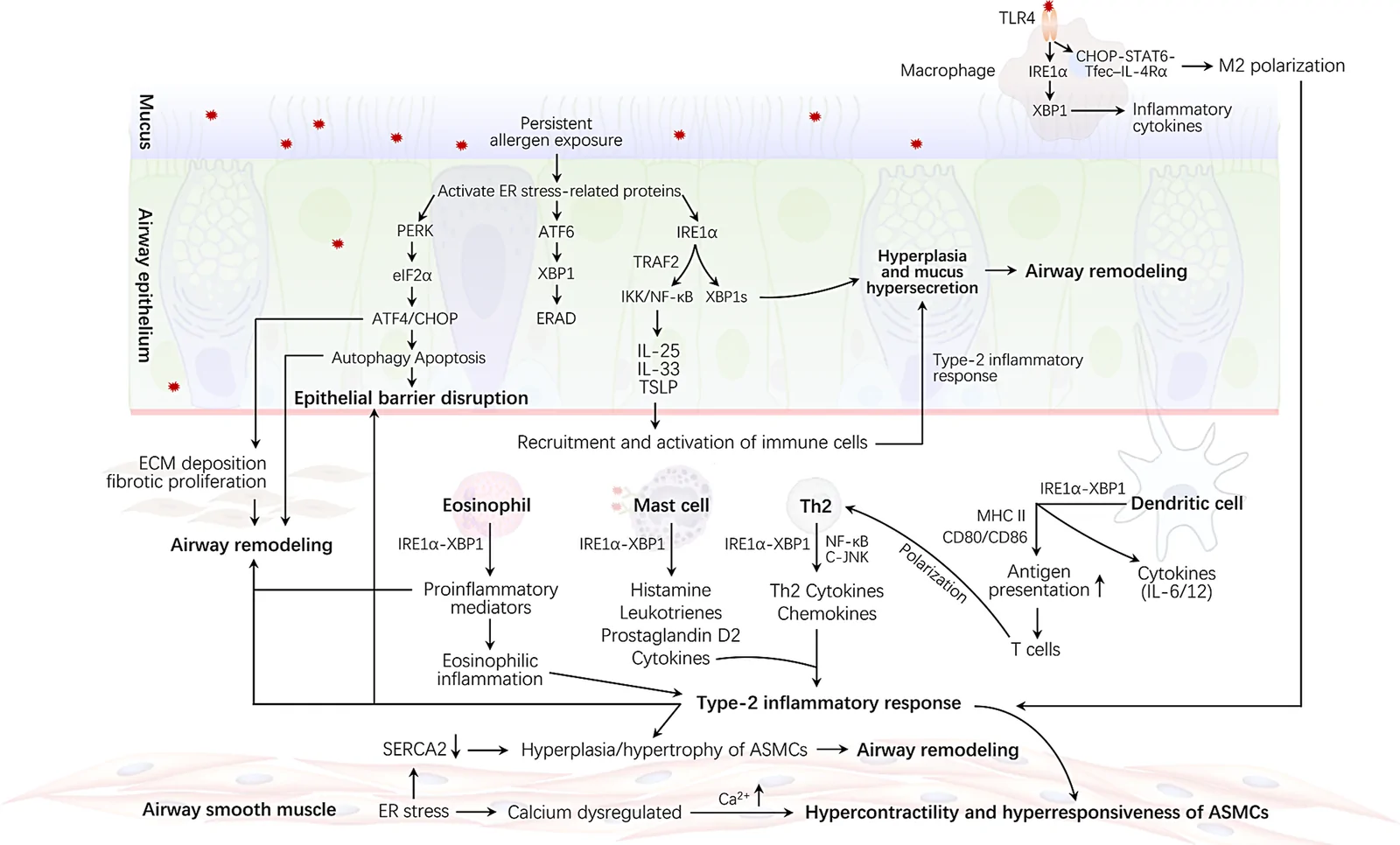
Schematic illustration of endoplasmic reticulum (ER) stress-mediated pathological cascades in allergic airway diseases (AADs).

#### Airway Epithelial Barrier Dysfunction and ER Stress

The airway epithelium acts as the first line of defense against inhaled allergens such as house dust mites and pollens, maintaining barrier integrity through tight junction proteins including zonula occludens-1 (ZO-1) and occludin [42, 43]. Allergen exposure directly damages airway epithelial cells, disturbs tight junction protein expression and localization, and impairs epithelial barrier integrity [44]. Such barrier breakdown correlates with the accumulation of unfolded/misfolded proteins in the ER lumen and subsequent ER stress [37]. ER stress sensors IRE1α and PERK activate UPR to restore ER homeostasis under mild stress [13, 45, 46]. However, persistent allergen exposure in AADs induces chronic ER stress, which further disrupts epithelial barriers. Activated PERK induces ER stress-associated apoptosis in airway epithelial cells through PERK-eIF2α-ATF4/CHOP Pathway, which further weakens the physical barrier and increases allergen permeability [20].

Activated IRE1α, via the IRE1α-TRAF2-IKK/NF-κB signaling pathway, contributes to the secretion of epithelial alarmins – including interleukin (IL)-25, IL-33, and thymic stromal lymphopoietin (TSLP) – that initiate and amplify type-2 immune responses [47]. This cytokine milieu recruits and activates downstream immune cells, thereby perpetuating airway inflammation. In addition, these cytokines disrupt epithelial barrier integrity, leading to a vicious cycle of increased epithelial permeability [48].

Therapeutic strategies targeting ER stress pathways, such as chemical chaperones, have demonstrated promise in restoring epithelial integrity and attenuating allergic inflammation, highlighting the critical role of ER stress in airway epithelial barrier dysfunction in AADs [49]. This forms a vicious cycle in which barrier disruption and ER stress reinforce each other, exacerbating allergic airway inflammation and remodeling.

#### Immune Cell Activation and ER Stress

ER stress not only affects structural airway cells but also modulates immune cell function, particularly dendritic cells (DCs), mast cells, macrophages, and lymphocytes – all of which are central to the pathogenesis of AADs. In DCs, ER stress modulates MHC class II molecule expression and costimulatory molecule (CD80/CD86) expression through the IRE1α-XBP1 axis, thereby strengthening antigen presentation and polarizing T cells toward a Th2 phenotype [50]. Polarized Th2 cells orchestrate type-2 immunity by secreting IL-4, IL-5, and IL-13, which drive IgE class-switching in B cells, eosinophil recruitment, goblet cell hyperplasia with mucus overproduction, and M2 macrophage polarization, thereby linking DC activation to allergic inflammation and tissue remodeling [50, 51]. The induction of XBP1s in DCs also leads to enhanced secretion of IL-6 and IL-12, further shaping the T-cell response.

Upon activation and degranulation, mast cells experience increased demands for protein synthesis and the secretion of mediators such as histamine, tryptase, prostaglandin D2, leukotrienes, and Th2-associated cytokines (IL-4, IL-5, IL-13, IL-9), imposing ER stress [5]. The UPR modulates the production and release of mast cell mediators through the IRE1α-XBP1s axis, thereby reinforcing the pro-inflammatory Th2 environment [52]. Mast cells localize near the submucosal mucous glands in the airways, and the release of mediators can trigger mucus hyperproduction by goblet cells.

Eosinophils are one of the most notable inflammatory cells observed in airway epithelium of AADs, which produce and secrete effectors such as eosinophil-derived neurotoxin and eosinophil peroxidase that directly generate reactive oxygen species and induce overall oxidative and ER stress. Eosinophils themselves depend on the IRE1α-XBP1 pathway for their activation and survival [53].

ER stress directly regulates T cell differentiation and function. XBP1s has been shown to promote Th2 differentiation and cytokine expression. This transcriptional regulation links ER stress signaling to the amplification of type-2 immunity [49, 54]. In addition, the IRE1α pathway activates TRAF2, recruiting IKK to stimulate the NF-κB pathway and ASK1 to stimulate c-JNK cascade, which amplifies Th2 cytokines (e.g., IL-4, IL-13) and chemokines that recruit eosinophils and mast cells. Th2 polarization subsequently stimulates the secretion of IL-4, IL-5, and IL-13, which trigger eosinophilic inflammation and IgE synthesis.

ER stress markers such as BiP and CHOP are robustly expressed in macrophages from nasal tissues of patients with allergic rhinitis, and these markers co-localize with plasma cells and eosinophils, indicating a broad involvement of the UPR in immune cell regulation under allergic conditions [55]. In an ovalbumin-induced allergic airway inflammation model, ER stress was shown to promote M2 macrophage polarization through a CHOP-dependent mechanism. Specifically, CHOP regulates STAT6 phosphorylation, which enhances the expression of transcription factor EC (Tfec); Tfec in turn transcribes IL-4 receptor α (IL-4Rα), thereby driving M2 polarization and sustaining the type-2 inflammatory response in AADs [56]. Collectively, ER stress serves as a pivotal regulator of immune cell activation and function, driving the type-2 inflammatory response in the airways.

#### Airway Remodeling and ER Stress

Airway remodeling, characterized by goblet cell hyperplasia, mucus hypersecretion, ASMC proliferation, and subepithelial fibrosis, is a hallmark feature of chronic AADs and contributes to irreversible airflow obstruction. Profibrotic mediators such as transforming growth factor-beta (TGF-β) induce ER stress in airway fibroblasts, activating UPR pathways that drive fibrogenesis [57]. ATF4 and CHOP upregulation further increases type I/III collagen expression, causing excessive extracellular matrix deposition [58, 59]. ER stress disrupts ER calcium homeostasis, as the ER serves as a major intracellular Ca^2+^ reservoir. Dysregulated Ca^2+^ signaling in ASMCs contributes to the hypercontractility and AHR [60]. Furthermore, deficiency of the sarco/ER Ca^2+^ ATPase-2 (SERCA2) in ASMCs has been demonstrated to promote their secretory and hyperproliferative phenotypes, which in turn promotes airway remodeling in asthma [61]. Persistent UPR signaling also induces autophagy and apoptosis in airway structural cells, altering cell survival and function, which further exacerbates airway tissue remodeling [62]. Experimental models have confirmed that pharmacological inhibition of ER stress using chemical chaperones such as 4-phenylbutyric acid (4-PBA), TUDCA, or TMAO attenuates key features of airway remodeling, including collagen deposition, mucus metaplasia, and AHR, indicating that sustained ER stress is a driving force underlying the structural changes in the airway [19, 45, 63]. XBP1s drives goblet cell hyperplasia and mucus hypersecretion by binding to the proximal region of the mucin 5B (MUC5B) promoter variant rs35705950, ultimately contributing to airway remodeling [56]. Thus, targeting ER stress and its downstream effectors represents a promising therapeutic strategy for preventing or reversing airway remodeling in AADs.

## Pharmacological Properties of TUDCA and Its Regulatory Mechanisms on ER Stress

### Chemical Chaperone Function and Maintenance of ER Homeostasis

Bile acids are hydroxylated steroids synthesized in the liver from cholesterol via sequential peroxisomal enzymatic reactions. They are subsequently conjugated with glycine or taurine (or sulfated) to improve solubility and facilitate their transport across hepatocyte canalicular membranes [64]. Ursodeoxycholic acid and its taurine-conjugated TUDCA are distinguished by strong hydrophilicity, which endows them with high bioavailability during enterohepatic circulation [65]. They constitute the main bioactive ingredients of bear bile. Synthetic TUDCA shares an identical structure with animal-derived TUDCA but achieves higher purity and is widely applied to treat bile acid metabolic disorders. Beyond hepatobiliary disorders, accumulating evidence has demonstrated that TUDCA exerts its core pharmacological effects by acting as a chemical chaperone within the ER [40, 64, 66, 67]. This function is critical for maintaining ER homeostasis, particularly by stabilizing the native conformation of proteins through hydrophobic interactions, thereby promoting the correct folding of nascent polypeptide chains in the ER lumen. The chemical chaperone activity of TUDCA reduces the burden of misfolded or unfolded proteins, which are the primary triggers of ER stress and the UPR. Treatment with TUDCA has been shown to significantly downregulate key ER stress markers, such as BiP, p-PERK, p-IRE1α, ATF6α, and CHOP, indicating effective attenuation of excessive UPR activation [19, 67]. For instance, in models of allergic airway inflammation, TUDCA administration mitigated ER stress-induced apoptosis and inflammation by reducing the expression of these markers, thereby protecting airway epithelial and immune cells from ER stress-mediated damage [19]. Furthermore, the chemical chaperone properties of TUDCA have been verified in various tissues [16, 68, 69], including retinal pigment epithelial cells, where it reduced oxidative damage and ER stress-induced apoptosis [16], and in renal mesangial cells, where it inhibited transforming growth factor-β1 (TGF-β1)-induced renal fibrosis by regulating ER stress [68]. These findings collectively highlight the role of TUDCA in stabilizing ER proteostasis, which is essential for cellular function and survival under stress conditions. By maintaining ER homeostasis, TUDCA not only prevents the accumulation of toxic protein aggregates but also modulates downstream signaling pathways involved in inflammation and apoptosis, making it a promising therapeutic agent for diseases characterized by ER stress, including AADs.

### Specific Regulation of Downstream UPR Signaling Pathways by TUDCA

TUDCA exerts selective modulation of the three canonical UPR branches – PERK, IRE1α, and ATF6 – thereby fine-tuning the cellular response to ER stress and preventing maladaptive outcomes such as apoptosis. Notably, TUDCA suppresses the overactivation of the PERK-eIF2α-ATF4-CHOP axis, the pathway critically involved in ER stress-induced apoptosis. TUDCA suppresses CHOP expression and nuclear translocation to shield airway epithelial and immune cells from ER stress-triggered apoptosis, a core pathological event in allergic airway inflammation and remodeling [46, 69, 70]. For the IRE1α-XBP1 pathway, TUDCA suppresses excessive signaling while retaining adaptive XBP1s-mediated transcription that maintains normal protein folding and secretion [71]. This balance is critical for goblet cell mucin production: TUDCA reduces pathological MUC5AC-driven mucus hypersecretion without impairing essential mucosal defenses [19]. Additionally, TUDCA modulates the ATF6 pathway by regulating the transcription of ER chaperones and lipid biosynthesis genes, contributing to the restoration of ER function and membrane integrity [46]. This comprehensive modulation of UPR branches by TUDCA ensures that the ER stress response remains adaptive rather than proapoptotic or pro-inflammatory. Experimental evidence from pulmonary fibrosis models has demonstrated that TUDCA pretreatment suppresses the Smad2/3-mediated epithelial-mesenchymal transition (EMT) and fibrosis by attenuating ER stress markers, including Bip and CHOP [72]. Similarly, in models of allergic airway disease, the inhibition of PERK and IRE1α phosphorylation is correlated with reduced airway inflammation and remodeling [49]. These findings highlight TUDCA’s ability to selectively target UPR signaling pathways, thereby mitigating the ER stress-associated pathological processes in AADs.

### Beyond ER Stress: Anti-Inflammatory and Antioxidant Effects of TUDCA

Aside from its ER chaperone activity, TUDCA possesses potent anti-inflammatory and antioxidant properties critical for AAD treatment. It blocks two major ER stress downstream inflammatory signaling pathways, NF-κB and c-JNK, both of which drive pro-inflammatory cytokine production. Suppression of these pathways lowers tumor necrosis factor-alpha (TNF-α), IL-6, and IL-8 secretion and mitigates airway inflammation [64, 73, 74]. Additionally, TUDCA mitigates mitochondrial dysfunction, which often occurs concurrently with ER stress via mitochondria-associated ER membranes (MAMs). It stabilizes mitochondrial membrane potential, decreases ROS generation, and thereby exerts potent antioxidant effects that protect cells against oxidative damage [75]. This mitochondrial protection is particularly crucial in AADs, where oxidative stress exacerbates airway inflammation and tissue remodeling. Furthermore, TUDCA modulates apoptotic pathways independent of ER stress, including death receptor-mediated apoptosis, thereby demonstrating multi-targeted cytoprotective properties [76, 77]. In preclinical models of allergic airway inflammation, TUDCA reduces eosinophilic infiltration and mucus hypersecretion by dampening oxidative stress and inflammatory responses [19]. Collectively, these anti-inflammatory and antioxidant actions of TUDCA complement its ER stress-regulatory functions, positioning it as a multifaceted therapeutic agent capable of addressing the complex pathophysiological mechanisms of AADs.

## Efficacy Evaluation of TUDCA in Preclinical Models of AADs

Preclinical mouse asthma models induced by ovalbumin or house dust mite strongly support TUDCA’s therapeutic efficacy against core asthmatic pathological phenotypes [19, 56, 78]. Administration of TUDCA via nasopharynx or oropharyngeal instillation in these models consistently alleviates AHR, a defining asthmatic trait. This functional improvement is accompanied by a marked reduction in inflammatory cell infiltration in the bronchoalveolar lavage fluid, particularly eosinophils, neutrophils, and lymphocytes, which serve as central mediators of airway inflammation. Histopathological analyses further confirm these findings, demonstrating that TUDCA treatment reduces the accumulation of inflammatory cells around bronchi and blood vessels, suppresses goblet cell hyperplasia, and decreases mucus overproduction, thereby alleviating mucus plugging and airway obstruction. Furthermore, TUDCA effectively inhibits collagen deposition and extracellular matrix remodeling – key drivers of irreversible airflow limitation in chronic asthma. At the molecular level, TUDCA downregulates ER stress markers, such as p-PERK and CHOP, indicating the mitigation of ER stress-induced apoptotic signaling pathways. Concurrently, TUDCA suppresses Th2 cytokines, including IL-4, IL-5, and IL-13, which regulate eosinophilic inflammation and IgE synthesis, while upregulating the expression of the anti-inflammatory cytokine IL-10, thereby restoring immune homeostasis. These multifaceted effects suggest that TUDCA exerts its protective effects by modulating both inflammatory and ER stress pathways, which are closely intertwined in the pathogenesis of asthma. Collectively, these preclinical data highlight TUDCA’s ability to ameliorate airway inflammation, hyperresponsiveness, and remodeling in allergic asthma models, supporting its potential as a novel therapeutic agent targeting ER stress and immune dysregulation in asthma.

## Translational Challenges and Future Perspectives

### Challenges from Animal Models to Clinical Application

The majority of current evidence supporting the therapeutic potential of TUDCA in AADs derives from studies using rodent models, predominantly mice [19]. Rodent models have helped clarify ER stress and UPR mechanisms in asthma, yet notable immunological and pathological differences exist between rodents and humans. Murine models cannot fully replicate the complexity and heterogeneity of human AADs in terms of immune cell composition, cytokine microenvironment, and airway remodeling [12, 13]. This gap underscores the need to validate TUDCA efficacy in humanized models or ex vivo human airway tissues before clinical testing. Furthermore, the optimal administration route of TUDCA for AADs remains undetermined. Systemic delivery may cause off-target effects and diminish pulmonary bioavailability, whereas local inhalation enhances lung-specific targeting. However, it requires formulation optimization to ensure stability and effective deposition in the lower airways [72]. In addition, the appropriate dosing regimen, therapeutic window, and long-term safety profile of TUDCA in chronic airway diseases (e.g., asthma) have not been systematically evaluated. Pharmacokinetic and toxicological studies are, therefore, essential to define these parameters, particularly given the chronic nature of AADs and the requirement for long-term sustained treatment. Another critical translational challenge is the remarkable heterogeneity of AADs, which encompass diverse endotypes defined by distinct inflammatory pathways and clinical phenotypes [79].

TUDCA has been evaluated in randomized controlled trials, phase II studies, and controlled clinical investigations across primary biliary cholangitis, liver cirrhosis, HCV-related chronic hepatitis, amyotrophic lateral sclerosis, transthyretin amyloidosis, and cholelithiasis [80–88]. These interventional studies consistently demonstrate a favorable safety profile, with oral capsule formulations administered at daily doses up to 2,000 mg and treatment durations extending to 2 years without significant adverse events. Nevertheless, whether these findings can be directly extrapolated to AADs remains unclear. Therefore, when positioning TUDCA as a potential therapeutic strategy for AADs, it is essential to fully recognize this critical evidence gap. Rigorous randomized controlled trials are needed to determine the safety, efficacy, optimal dosing regimen of TUDCA in patients with AADs before any definitive conclusions regarding its clinical utility can be drawn.

### Development of Novel Formulations and Delivery Systems

To improve pulmonary targeting and efficacy while reducing systemic exposure, researchers are developing advanced TUDCA delivery systems. Nanotechnology-based formulations such as liposomes and polymeric nanoparticles [89, 90] represent promising vehicles for TUDCA encapsulation, improving its solubility, stability, and controlled release within the lung microenvironment. Liposome-loaded TUDCA enables efficient aerosolization and deep lung deposition to elevate local drug concentration at the inflamed, ER stress-affected airway lesions. Similarly, dry powder inhalers incorporating TUDCA-loaded nanoparticles are being explored to provide patient-friendly, non-invasive administration with prolonged pulmonary retention time. Beyond formulation optimization, chemical modification strategies – such as prodrug design or the synthesis of TUDCA analogs – aim to improve membrane permeability, metabolic stability, and selective uptake by target cells, including activated immune cells and airway structural cells undergoing remodeling [91]. These modifications could potentiate intracellular delivery to the ER, thereby augmenting its chaperone activity and capacity to mitigate ER stress. However, the development of these novel formulations necessitates rigorous preclinical evaluations to assess their pharmacodynamics, pharmacokinetics, safety profiles, and potential immunogenicity. Overall, advances in drug delivery technologies and chemical modification strategies hold significant promise for optimizing TUDCA’s clinical utility in AADs by enhancing lung-specific targeting, bioavailability, and therapeutic potency.

### Combinatorial Strategies Integrating TUDCA with Existing Treatments

Therapeutic strategies combining TUDCA with established agents are emerging as a compelling area of investigation. These combinations capitalize on TUDCA’s pleiotropic mechanisms, including the attenuation of ER stress, suppression of apoptosis, and resolution of oxidative and inflammatory injury. Such multi-pronged interventions not only achieve synergistic therapeutic gains exceeding monotherapy thresholds but also permit lower dosing of primary pharmacologics, effectively decoupling efficacy from toxicity.

Glucocorticoids (GCs) serve as the primary first-line treatment for AADs, broadly suppressing Th2 inflammation through inhibition of NF-κB and pro-inflammatory transcription programs. Nevertheless, long-term GC monotherapy leads to prominent drawbacks, including irreversible airway epithelial damage and widespread steroid refractoriness in severe asthma, which greatly limits clinical outcomes. You et al. [92] found that sustained dexamethasone exposure excessively activates the PERK-eIF2α-CHOP cascade in airway epithelial cells, triggering epithelial apoptosis, degrading tight junction proteins ZO-1 and occludin, and boosting the release of type 2 alarmins IL-25, IL-33, and TSLP. This GC-amplified maladaptive UPR further impairs epithelial barrier integrity, increases allergen infiltration, and drives steroid insensitivity in severe AADs. As a potent chemical chaperone, TUDCA specifically blocks GC-induced PERK-CHOP hyperactivation, stabilizes epithelial structure, and offsets GC-mediated mucosal injury. Co-administration of TUDCA with low-dose GCs, therefore, represents an ideal adjuvant regimen that retains the broad anti-inflammatory activity of corticosteroids while alleviating their intrinsic toxic epithelial effects [92].

The synergistic therapeutic superiority of TUDCA-based combination regimens has been validated across multiple disease models. For instance, in diabetic acute kidney injury, TUDCA specifically targets the BiP/PERK/CHOP cascade to resolve maladaptive ER stress, while phloretin, a natural Toll-like receptor (TLR4) pathway inhibitor, blocks the TLR4/NF-κB inflammatory axis. Pairing TUDCA with phloretin can simultaneously interrupt the bidirectional crosstalk between ER stress and inflammation, achieving stronger cytoprotection [93]. Although demonstrated in diabetic kidney injury, this dual-axis logic – simultaneously braking maladaptive ER stress and the downstream inflammatory cascade – provides a translatable blueprint for designing synergistic regimens in AADs, particularly for patients with refractory type-2 inflammation.

## Conclusion

ER stress acts as a pivotal pathological hub linking allergen exposure, immune cell activation, airway epithelial damage, and airway remodeling in AADs. TUDCA, a well-characterized ER stress alleviator, opens a promising therapeutic avenue for AADs by simultaneously restoring ER proteostasis, suppressing maladaptive UPR signaling, and counteracting oxidative and inflammatory injury. While current preclinical evidence supports its efficacy in mitigating airway inflammation, hyperresponsiveness, and structural remodeling, several critical limitations warrant attention to guide future translation.

First, nearly all existing mechanistic and efficacy data derive from rodent AAD models, which fail to recapitulate the full spectrum of human AAD heterogeneity – including diverse endotypes (e.g., Th2-high vs. Th2-low asthma), variable remodeling patterns, and steroid-refractory subtypes. Future studies should validate TUDCA’s effects in human precision-cut lung slices, induced pluripotent stem cell-derived airway models, and well-phenotyped patient cohorts to establish translational relevance.

Second, key pharmacological parameters remain undefined for chronic AAD management. The optimal route of administration (systemic vs. inhaled), dose-response relationship, long-term safety profile, and therapeutic window require systematic evaluation, particularly given the need for sustained treatment in chronic airway diseases. Inhaled nano-formulations (liposomal, polymeric nanoparticle-based carriers) and TUDCA analogs with enhanced lung tropism represent priority areas for formulation optimization to maximize pulmonary bioavailability while minimizing off-target effects.

Third, rational combinatorial strategies merit deeper exploration. Preclinical data suggest TUDCA can offset GC-induced epithelial toxicity by inhibiting PERK-CHOP hyperactivation; extending this work to test synergies with biologics targeting type-2 inflammation (e.g., anti-IL-4/IL-13, anti-TSLP antibodies) or with agents blocking ER stress-inflammation crosstalk (e.g., TLR4 inhibitors) could yield more potent, lower-toxicity regimens for refractory AADs. Mechanistic studies should also clarify whether TUDCA exerts cell-type-specific effects across airway epithelial cells, immune subsets, and structural cells during different disease stages.

Finally, rigorous large-scale long-term clinical trials are indispensable to validate TUDCA’s long-term safety and efficacy in humans, laying a solid foundation for the clinical transformation of this candidate agent and ultimately improving the quality of life of patients suffering from chronic allergic airway disorders. By integrating basic pathological research with translational innovation, future work on TUDCA has the potential to shift AAD management from symptom control to targeted modulation of core pathogenic drivers, ultimately improving outcomes for patients with these chronic, often disabling conditions.

## Conflict of Interest Statement

The authors have no conflicts of interest to declare.

## Funding Sources

This research did not receive any specific grants from any funding agency in the public, commercial, or not-for-profit sectors.

## Author Contributions

Yining Han conceived the review and wrote the paper.
